# The neurodynamics of epilepsy: a homotopy analysis between current-based and conductance-based synapses in a neural field model of epilepsy

**DOI:** 10.1186/1471-2202-16-S1-P23

**Published:** 2015-12-18

**Authors:** Andre DH Peterson, Iven MY Mareels, Hamish Meffin, David B Grayden, Mark J Cook, Anthony N Burkitt

**Affiliations:** 1 NeuroEngineering Lab, Dept. of Electrical & Electronic Engineering, University of Melbourne, Melbourne, Australia; 2 Department of Medicine, University of Melbourne, Melbourne, Australia; 3 Centre for Clinical Neurosciences, St. Vincent's Hospital, Melbourne, Australia; 4 NVRI, Melbourne, Australia; 5 Bionics Institute, Melbourne, Australia

## Introduction/Background

Unlike Hodgkin-Huxley type spiking models, the overwhelming majority of neural field models use current-based synapses [1]. Although there exist neural field models that employ conductance-based synapses, it is not clear what their exact effects on the dynamics are, particularly with respect to epileptic dynamics. Neural field models of epilepsy typically describe the transition to seizure-like activity as a bifurcation [2]. This research examines the effects of conductance-based synapses on the transition from normal to seizure-like activity in neural field models.

## Methods

In this research we construct a neural field model with a homotopy parameter, κ, such that when κ = 0, then the model has current-based synapses, and when κ = 1, it has conductance-based synapses. This enables us to compare and explain the key differences in dynamics caused by the different synaptic mechanisms. In particular we perform a bifurcation analysis using the homotopy parameter, κ, as a bifurcation parameter to rigorously analyse both models.

## Results

We find that the conditions under which bifurcations take place are quite different for each synaptic mechanism. The feedback term from the membrane potential, which makes conductance-based synapses nonlinear, considerably affects the dynamics. This is especially so in comparison with their linear current-based counterpart. In the current-based model increasing the external input parameter in conjunction with the network balance parameter generates a Hopf bifurcation, which is typically interpreted as a transition to a seizure-like state [2]. In comparison, changing these two parameters in the conductance-based model has no effect due to the feedback from the membrane potential term. The suppression of the transition to a seizure-like state occurs at a critical value of the homotopy parameter κc.

## Discussion/Conclusion

In terms of neural modeling of epilepsy, this demonstrates that the new features introduced in the more biophysically realistic conductance-based synapses act as an anti-epileptic regulatory mechanism that suppresses the transition to seizure. The homotopy parameter, κ, is interpreted as being proportional to the synaptic background activity that affects the conductance state of neurons and results in fluctuations of their membrane potentials [3]. It is these fluctuations that suppress the transition to seizure-like activity and need to be taken into account in neural models. If these fluctuations are of reasonably small amplitude, for example, as in resting state behaviour, then current-based synapses can be an adequate approximation. However, if these fluctuations are larger in amplitude, for example, as in oscillatory or seizure-like activity, then they need to be included [4]. Essentially, we have constructed a mathematical mapping between two different synaptic mechanisms and examined their effects on the dynamics of a typical neural field model. This calls into question previous results of neural field models that use current-based synapses, including those of epilepsy, as using a more biophysically realistic synaptic mechanism yields significantly different results.
